# State estimation in an islanded hybrid solar-wind DC microgrid using Unscented Kalman Filter

**DOI:** 10.1016/j.heliyon.2025.e42073

**Published:** 2025-01-17

**Authors:** Nima HajiHeydari Varnoosfaderani, Amir Khorsandi

**Affiliations:** Department of Electrical Engineering, Amirkabir University of Technology, Tehran, Iran

**Keywords:** Islanded DC microgrid, Microgrid modeling, State estimation, Non-linear state estimation, Renewable generation, Kalman filter, Unscented Kalman filter

## Abstract

This paper investigates the state estimation problem for an islanded DC microgrid. The microgrid consists of energy storage units, as well as wind and solar generation units, all designed to supply power to a variable DC load, specifically an electric vehicle charging station. To ensure accurate state estimation, a third-order model is utilized for the battery storage system. A DC/DC boost converter is implemented for the photovoltaic system, resulting in a second-order state-space model. The wind generation unit, which incorporates a three-phase synchronous generator, is represented by a fourth-order nonlinear state-space model. Consequently, the entire microgrid is described by a ninth-order nonlinear state-space model, which is simplified by excluding two states from the battery model. This study also includes a comprehensive analysis of the load's dynamic modeling and behavior. A second-order state-space model is derived for the electric vehicle charging station as the load. A variant of the Kalman filter is employed as the state estimation algorithm, which is model-based. The algorithm is implemented to estimate the unknown or unmeasurable states of the microgrid, incorporating known inputs including load, solar irradiation, wind speed, and photovoltaic panel temperature. The algorithm employs an unscented transform to eliminate the need for system linearization, with the Kalman filter playing a pivotal role in the estimation process. The robustness of this convergence is evaluated under three scenarios: first, when encountering sudden load variations; second, when improperly initializing the estimation process; and third, during abrupt changes and noisy measurements in solar irradiation, one of the system inputs. Simulations conducted in MATLAB/SIMULINK demonstrate that the algorithm's estimations converge to actual values with a satisfactory level of accuracy and precision within a desirable time interval.

## 1 Introduction

In recent decades, there has been widespread development in renewable power generation (REG), leading to the emergence of smart microgrids (MGs) as one of its most popular outcomes. Due to the stochastic nature of renewable energy sources, efficient energy management systems (EMS) are essential for ensuring a steady and optimal operation of different parts of the MG. In other words, EMSs must be capable of addressing uncertainties to safeguard the system against unexpected issues such as overcharging or discharging of energy storages, unbalanced power flow, etc. These uncertainties include model uncertainties, measurement uncertainties, and uncertainties arising from various noise sources affecting the system [1]. Additionally, sudden changes in wind speed or solar irradiation may necessitate the need for an efficient and robust EMS to effectively respond and uphold the performance quality of the MG.

Therefore, it is crucial to dynamically and robustly track the states of the renewable energy-based MG, particularly when it is integrated into the primary power grid. Additionally, understanding the states of the system is a fundamental requirement for the operation of the MG's basic functionalities and for responding to feedback signals [2]. Consequently, the estimation of system states is a crucial task that aims to ensure the normal and secure operation of the MG [3]. However, various challenges can arise in measuring and monitoring the states of the system. For example, model predictive control of inverters necessitates efficient flux observers [4], and estimating the state of charge in batteries is complex due to the inability to directly measure this quantity [5]. In other words, the state estimation (SE) algorithms are essential in situations where a reliable and accurate measurement systems and equipment are unavailable. Various estimation algorithms have been employed in power system studies including the weighted least-square method [6], particle filters [7,8], distributed estimators [2], H-infinity methods [9], etc. These methods have been applied in a range of power system studies including photovoltaic (PV) generation powered AC/DC hybrid MG [10], integration of IoT networks and SE algorithms in an unstable MG [11], using distributed estimator for designing islanded [12] or networked [13] MGs, developing operation and protection schemes for large and high power MGs, as well as employing SE algorithms in wind farms to estimate active and reactive power flows and important states of each generator [14]. It is important to highlight that certain state estimation algorithms operate at regular, albeit relatively lengthy, intervals under the assumption that the power system remains in a quasi-steady state. This assumption, however, may not hold true for power systems incorporating renewable energy resources, which introduce uncertainties and dynamic elements [2]. Furthermore, in most of the mentioned methods, the target system is assumed to be linear. However, it is rare to find a system that is completely unaffected by linearization, which may lead to malfunctions and divergence in these algorithms. On the other hand, linear methods provide a significant advantage in terms of mathematical and computational simplicity compared to other state estimation methods.

The Kalman filter (KF) has played a significant role in estimation theories since its introduction in the 1960s [15], establishing itself as the primary estimation algorithm. Initially tailored for linear systems, numerous adaptations have since been developed to augment its performance for both linear and nonlinear systems. The extended Kalman filter (EKF) stands as the first significant variant specifically designed for nonlinear systems, utilizing the Taylor series expansion to linearize the nonlinear system around its operating point. As a result of the accumulation of errors caused by linearization, an alternative version of the Kalman filter was developed: the Unscented Kalman Filter (UKF). This nonlinear adaptation of the Kalman filter is based on the simple principle that "it is easier to approximate a probability distribution than to approximate an arbitrary nonlinear function or transformation" [1]. In order to simplify the nonlinear estimation process, the UKF propagates the probability density function (PDF) using a set of "sigma points". This propagation enhances the estimation accuracy up to second order.

The KF family has been widely applied to power system studies and state estimation in recent years. The unscented variant has proven effective in applications involving induction machines, gas turbines, and fuel cells [1]. Moreover, the UKF has been highly successful in designing control strategies for DFIG wind turbines interconnected with complex power networks [16]. Similarly, the extended variant has been developed and utilized in various scenarios, including sensorless speed control of induction [17] and synchronous [18] machines. Additionally, the EKF has been applied to conduct dynamic state estimation for a wind turbine, which is represented by a 16th-order model [19]. The KF family has also been extensively employed in various MG studies, such as MG stabilization [20], control of induction generator wind energy DC MGs using an artificial neural network (ANN) [21], and fault detection in DC MGs [22]. The traditional variant has also been applied for energy theft detection in islanded MGs [23].

MGs based on REGs exhibit a significant level of nonlinearity and uncertainty, making UKF and EKF suitable and powerful tools for these situations. It is important to note that KF methods typically require an appropriate model that accurately accounts for dynamics. On the contrary, the traditional WLS method [24], widely employed in the power systems arena, generally assumes the system states to be static or changing slowly in relation to the system's time constants.

Previous models of microgrids in the literature exhibit several weaknesses. For instance Ref. [25], considers only bus voltages and currents as state variables, overlooking other critical states in the system, such as rotor angle and battery state of charge. The 12th-order model presented in Ref. [26] is overly complex for real-time implementation given current computational capacities, and it also neglects the inclusion of wind generation units and battery storage systems. Furthermore, although the model presented in Ref. [27] considers diesel generators and induction machines, it is linearized around a specific point of operation which may limit its applicability for state-space modeling [28,29]. Other studies on microgrid modeling have similarly neglected key components. For example, the model in Ref. [30] lacks storage units, the model in Ref. [31] does not include any generators, and the model in Ref. [32] omits PV units. The algorithm presented in this paper is inspired by Ref. [33], where the UKF algorithm is employed for fault detection in a grid-connected microgrid.

The main objective of this paper is to develop an accurate and robust UKF estimator that can handle sudden changes in power demand effectively. The main contributions of this paper are as follows.

• **Accurate Model for Estimation Algorithms**: Given the importance of an accurate model in model-based estimation algorithms such as the KF, this paper presents an integrated nonlinear 11th-order state-space model for a standalone DC hybrid wind-solar MG with battery energy storage, incorporating wind speed, solar irradiation, and load power as the primary system inputs.
• **Model Flexibility**: The proposed model is flexible and can be simplified to a 9th-order model without compromising system performance or estimation accuracy, making it suitable for real-time applications.
• **Subsystems and Decoupling**: The model consists of four subsystems: load, storage, solar, and wind. Power flow in the grid serves as the linking variable. By selecting an appropriate output to measure from the model, the system can be decoupled into three distinct fields during the estimation process: one for the storage system, another for the subsystem containing solar and wind generation units, and the last for the load side.
• **Parallel Computing Implementation**: This decoupling facilitates parallel computing implementation, allowing each field to operate independently at any given time instant. This independence permits different sampling times for each field, meaning that the field with the longer sampling time does not need to undergo an estimation process that operates at a shorter sampling time, significantly reducing computational effort.

The remainder of the paper is organized as follows: In Section 2, the non-linear state-space models of the MG components are presented. Section.3 provides a brief explanation about the main UKF estimation algorithm. Section 4 presents the implementation of the estimation algorithm on the DC MG. In Section 5, the simulation method is explained, and results are presented. Finally, the conclusions are given in section 6.

## 2 Modeling and problem formulation

**Fig. 1:**
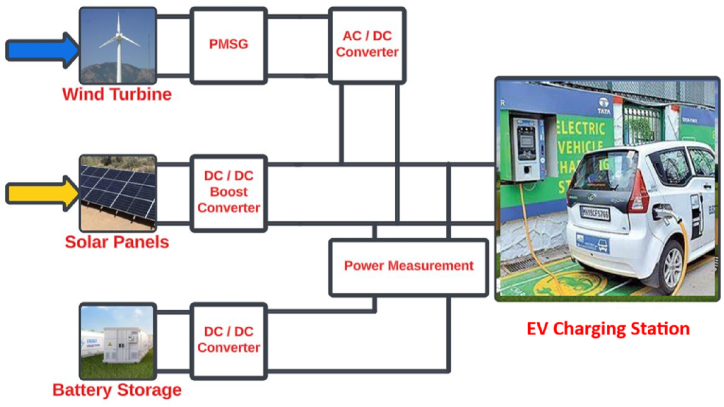
The DC microgrid and its subsystems.

Fig. 1 shows the diagram of the MG under study. As previously mentioned, the DC MG consists of four main interconnected subsystems using power conversion tools. An Electric Vehicle (EV) charging station is considered as a load. To ensure effective and accurate estimation, each component should be represented by an equivalent state-space model, despite many having nonlinear models. Previous studies often linearize these models around a specific operating point, especially in controller designs. The modeling and simulation approach presented in this paper is based on two primary assumptions: the first is that there is a superior controller that provides benchmarks for the local controllers to achieve [34]. The second assumption is the proper sizing of each component of the MG to ensure a feasible power balance [26].

### 2.1 Battery model

The primary aim of incorporating batteries, or other energy storage systems, is to maintain power balance within the MG. Among the different options available, Lithium-ion batteries are the most commonly used for MG applications; therefore, only this type of battery is considered in this paper. Given the use of model-based state estimation algorithms, it is crucial to model and evaluate battery performance to comprehend their behavior in MG systems through a combination of experiments and simulations [5].

Nonlinear battery models are generally categorized into four main types. The simplest model is the Rint model [35], which includes only a voltage source and a resistor. However, this model only accounts for the static state of the battery and is not suitable for dynamic state estimation. On the other hand, the GNL [36] and PNGV [35] models offer accurate simulation of battery behavior in various situations. Nonetheless, they are complex and challenging to implement, and may be prone to instability due to error propagation.

**Fig. 2:**
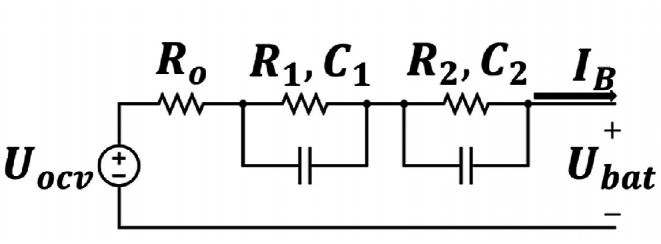
Battery 2-RC model.

The N-RC model demonstrates satisfactory performance in dynamic real-time simulations. This model comprises N RC branches connected in series, along with an internal resistor and a voltage source. To accurately capture the chemical behavior of the battery and establish an analytical relationship between open-circuit voltage and state of charge (SOC), parameter estimation is necessary for this model. As stated in Ref. [37], for a balance of simplicity and accuracy in battery simulation, N is set at 2. Fig. 2 shows the circuit equivalent of the 2-RC model, with its corresponding equations given by equations (1), (2).

(1) $${\dot{U}}_{1}=\frac{i\left(t\right)}{C_{1}}-\frac{U_{1}}{R_{1}C_{1}}$$

(2) $${\dot{U}}_{2}=\frac{i\left(t\right)}{C_{2}}-\frac{U_{2}}{R_{2}C_{2}}$$

Where $U_{j}$ is the voltage drop along the j-th RC branch.

The output voltage of the battery is described by equation (3).

(3) $$U_{bat}=U_{ocv}-U_{1}\left(t\right)-U_{2}\left(t\right)-i\left(t\right)R_{o}$$

We can also write equations (4), (5) for the SOC:

(4) $$SOC\left(t\right)={SOC}_{0}-\int_{0}^{t}\frac{\eta \times i\left(t\right)}{Q_{c}}dt$$

(5) $$\dot{SOC}=-\frac{\eta \times i\left(t\right)}{Q_{c}}$$

Where η is the coulomb efficiency and $Q_{c}$ is the maximum useable capacity of the battery.

The state-space model of the battery comrises three equations, (1), (2), and (5), as state equations. The measurable output of the battery is also described by equation (3).

In equation (3), $U_{ocv}$ is determined through diffrent methods of parameter identification, which results in equation (6).

(6) $$U_{ocv}=f\left(SOC\right)$$

Although the state equations ((1), (2), and (5)) are linear, the output equation described by equation (3) is highly nonlinear due to the presence of polynomial terms in equation (6). It should be noted that it is more common to represent the relationship between OCV and SOC in polynomial form rather than in various other mathematical forms.

In this study, the measured (and linking) output is power, which can be easily obtained by multiplying the output current and voltage. Therefore, equation (3), as the output equation of the state-space model, can be converted to represent the net output (or input) power of the battery.

### 2.2 PV-array and converter model

**Fig. 3:**
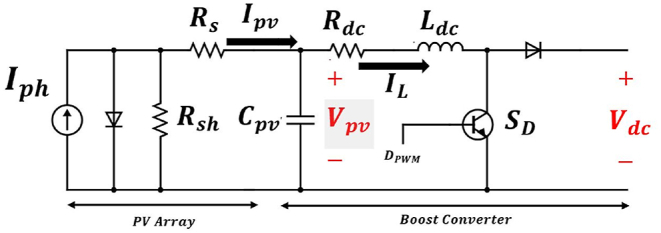
PV-array and Boost converter equivalent circuit.

In MG studies, PV arrays are commonly represented using the integrated equivalent circuit known as the PV single diode model. These PV arrays are connected to the main grid through a DC/DC boost converter, which is used to regulate the output voltage within a specified range. It should be noted that buck or buck-boost converters may also be utilized in such MGs, which are state-space modeled in Ref. [38]. Fig. 3 illustrates the PV array and its boost converter connected to the main grid (DC link).

#### 2.2.1 PV-array

The PV-array section is characterized by several parameters. The photocurrent, $I_{ph}$, is a function of solar irradiance, G, and cell temperature, T. Additionally, it includes two resistive branches: $R_{s}$ represents the series resistance, and $R_{sh}$, represents the shunt resistances [39]. Although these resistances are sometimes assumed to be constant, a more accurate model describes them as functions of irradiance and cell temperature [40].

By writing the algebric circuit equations of the PV-array section, equation (7) can be derived.

(7) $$I_{pv}=I_{ph}-I_{s}\;\left(e^{\frac{V_{pv}+I_{pv}\;R_{s}}{a}}-1\right)-\frac{V_{pv}+I_{pv}\;R_{s}}{R_{sh}}$$

It is nearly impossible to find an analytical solution for this equation. On the other hand, employing numerical methods would result in increased computational effort and raise the risk of error propagation. It is demonstrated in Ref. [39] that equation (7) can be rewritten in a different form using the Lambert W function:

(8) $$I_{pv}=\frac{R_{sh}\;I_{ph}-V_{pb}}{R_{s}+R_{sh}}-\frac{a}{R_{s}}\;W\left\{\frac{R_{s}\;R_{sh}\;I_{s}}{a\;\left(R_{s}+R_{sh}\right)}\;e^{\frac{R_{sh}\;\left(R_{s}\;I_{ph}+V_{pv}\right)}{a\;\left(R_{s}+R_{sh}\right)}}\right\}$$

The major benefit of equation (8) is that it allows for the expression of $I_{pv}$ as a function of $V_{pv}$. The calculation of the PV-array parameters is detailed in Ref. [40].

#### 2.2.2 Boost converter model

Boost converters employ varying duty cycle, *D*, to maintain the output voltage within a certain range. These switching circuits are typically represented by their average model. The state-space model of the boost converter is described by the following equations [39]:

(9) $${\dot{V}}_{pv}=\frac{\left(I_{pv}-I_{L}\right)}{C_{pv}}$$

(10) $${\dot{I}}_{L}=\frac{\left(V_{pv}-R_{dc}\;I_{L}-\left(1-D\right)V_{dc}\right)}{L_{dc}}$$

where $C_{pv}$, $R_{dc}$, $L_{dc}$ are the parameters of the converter. In short time intervals and in the absence of sudden changes in irradiance, $V_{dc}$ and duty cycle, D, can be assumed to be constant. Equations (9), (10) seem to be linear; however, according to equation (8), $I_{pv}$ in equation (9) is highly non-linear, which causes nonlinearity into the model.

### 2.3 Wind generation model

#### 2.3.1 Wind turbine model

The wind generation section consists of a wind turbine (WT) and typically includes a permanent magnet synchronous generator (PMSG) that is connected to the main grid through an AC/DC converter. The output power of the WT ($P_{wt}$) is related to the wind speed (v) by the following equation [41]:

(11) $$P_{wt}=0.5\;\rho \;A\;v^{3}\;C_{p}\left(\beta ,\lambda \right)$$

where A is the swept area by the rotor blades and ρ is the air density. The capacity factor of the WT ($C_{p})$ in equation (11) is determined by equation (12).

(12) $$C_{p}=0.5176\;\left(\frac{116}{\lambda_{i}}-0.4\;\beta -5\right)e^{\frac{-21}{\lambda_{i}}}+0.0068\;\lambda$$

The tip-speed ratio (TSR) λ is the ratio between the rotor blade tip speed and the wind speed. This dimensionless parameter is commonly used to characterize the efficiency of a wind turbine. $\lambda_{i}$ is defined by:

(13) $$\frac{1}{\lambda_{i}}=\frac{1}{\lambda +0.08\;\beta }-\frac{0.035}{\beta^{3}+1}$$

In equation (13). the rotor blade pitch angle, denoted as β, is actively controlled to track the reference rotor speed $\omega_{wt}$, ensuring stable operation of the wind turbine. This control loop helps prevent instability in the wind turbine. The detailed description of the pitch angle control method can be found in Ref. [42].

#### 2.3.2 PMSG model

There are various models of different orders for PMSGs. While a second-order model is appropriate for stability analysis, higher-order models, such as sixth- or seventh-order models, are utilized in specific scenarios, such as parameter identification problems. In this article, a fourth-order model of the PMSG is employed, offering a balance between accuracy and computational effort. This model is formulated in the per-unit system and the dq0 domain as shown in equations (14), (15), (16), (17).

(14) $$\dot{\delta }=\omega_{0}\;\Delta \omega$$

(15) $$\dot{\Delta \omega }=\frac{1}{J}\;\left(T_{m}-T_{e}-D\;\Delta \omega \right)$$

(16) $${\dot{e}}_{q}^{\prime }=\frac{1}{T_{d0}^{\prime }}(E_{fd}-e_{q}^{\prime }-\left(x_{d}-x_{d}^{\prime }\;\right)i_{d}$$

(17) $${\dot{e}}_{d}^{\prime }=\frac{1}{T_{q0}^{\prime }}(-e_{d}^{\prime }+\left(x_{q}-x_{q}^{\prime }\;\right)i_{q}$$

where ω and $\omega_{0}$ are the instantanous and synchronous speeds, respectively. $T_{m}$ is the mechanical torque. J and D denote the inertia and damping coefficient of the rotor, respectively. $E_{fd}$ is the internal voltage of armature. $e_{q}^{\prime }$ and $e_{d}^{\prime }$ represent the transient voltage of the q-axis and d-axis, respectively. Additionally, $T_{q0}^{\prime }$, $T_{d0}^{\prime }$, $x_{d}$, $x_{d}^{\prime }$, $x_{q}$ and $x_{q}^{\prime }$ are parameters of the PMSG defiend in Ref. [43].

The air-gap torque $T_{e}$ is equal to the electrical power when neglecting the stator resistance and assuming $\omega_{r}$ is 1.0 p.u. This can be formulated as:

(18) $$T_{e}=e_{d}i_{d}+e_{q}i_{q}$$

(19) $$e_{d}=V_{t}\;\sin\;\delta$$

(20) $$e_{q}=V_{t}\;\cos\;\delta$$

(21) $$i_{d}=\frac{e_{q}^{\prime }-V_{t}\;\cos\;\delta }{x_{d}^{\prime }}$$

(22) $$i_{d}=\frac{V_{t}\;\sin\;\delta }{x_{q}}$$

where $e_{d}$, $e_{q}$, $i_{d}$, $i_{q}$ are the d-axis and q-axis voltages and currents, respectively. $V_{t}$ is the terminal voltage. Replacing equations (19), (20), (21), (22) into equation (18) results in the following equation:

(23) $$P_{out}=\frac{V_{t}}{x_{d}^{\prime }}\;e_{q\;}^{\prime }\;\sin\;\delta +\frac{V_{t}^{2}}{2}\;\left(\frac{1}{x_{q}}-\frac{1}{x_{d}^{\prime }}\right)\sin\;2\;\delta$$

Equations (14), (15), (16), (17) represent the state equations, and equation (23) encapsulates the measurable output of the PMSG state-space model. Further description on the parameters of this model can be found in Ref. [43].

#### 2.3.3 AC/DC converter

As mentioned in the introduction, the power flowing in the grid acts as the linking variable between these subsystems. The output power of the PV array passing through the boost converter is obtained by multiplying the instantaneous voltage and current, as described by equations (9), (10). The method for calculating the power of the battery is also detailed in the first subsection of this section. The output equation of the PMSG state-space model also provides its output power for analysis. Power converter tools, including inverters and converters, are highly efficient devices capable of achieving over 90 % efficiency at their nominal point [44]. This high efficiency enables us to overlook their impact on power flow resulting from power loss and to assume that the power generated by the PMSG is directly injected into the grid. A similar assumption is applied to the DC/DC converter connecting the battery to the main grid.

However, the presence of the DC/DC boost converter is necessary due to the algebraic nature of the equations describing the PV-array (equation (8)), which is not suitable for dynamic purposes. In contrast, the equations of the PMSG and the battery are dynamic in nature. Incorporating the boost converter introduces the dynamic equations which also include control signals (duty cycle D). In essence, this results in a dynamic system where irradiation and cell temperature act as the system input, and the duty cycle serves to represent the control loops. Moreover, the boost converter can be integrated into a set of differential equations ((9) and (10)) that describe the system's dynamics.

### 2.4 Load side model

**Fig. 4:**
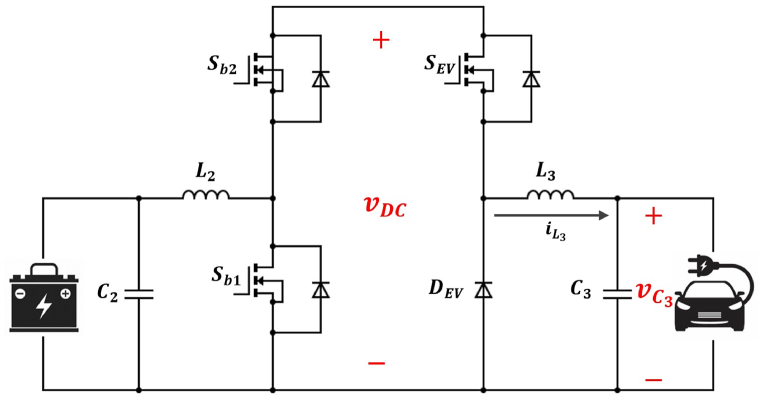
EV charging equivalent circuit.

When an EV is connected to the load bus of the MG, the charging process can occur through a storage unit, directly from generation units (which is rare), or a combination of both generation and storage units [45]. Assuming that charging is primarily from the storage unit, the equivalent circuit is depicted in Fig. 4. In this situation, switches $S_{b1}$ and $S_{b2}$ are turned off while $S_{EV}$ is turned on. Using circuit analaysis, we have:

(24) $$L_{3}\frac{di_{L3}}{dt}=v_{DC}-v_{C3}$$

(25) $$C_{3}\frac{dv_{C3}}{dt}+\frac{v_{EV}}{R_{EV}}=i_{EV}$$

where $v_{DC}$, $v_{EV}$ and $i_{EV}$ represent the DC link voltage, the charger voltage and EV load, respectively. $i_{L3}$ consists of two components: the current of capacitor $C_{3}$ and the current flowing to the EV. Assuming that the current from $C_{3}$ is negligible, $i_{L3}$ can be considered equivalent to $i_{EV}$. As previously stated, one of the system inputs is the load, which is derived from the multiplication of the $i_{EV}$ and $v_{DC}$. Therefore, by dividing the load by the link voltage, the EV charging current (which is the input in equation (25)) can be determined. Upon rewriting equations (24), (25), it is observed that they bear similarity to those derived for the boost converter state-space model (equations (9), (10)).

### 2.5 Problem formulation

#### 2.5.1 Generation and storage side

By arranging the state equations of each subsystem, the objective is to determine the state vector represented as equation (26), with a high level of accuracy and rapid convergence:

(26) $$x=\left[\begin{array}{ccccccccc} SOC & U_{1} & U_{2} & V_{pv} & I_{L} & \delta & \Delta \omega & e_{q}^{\prime } & e_{d}^{\prime } \end{array}\right]$$

The measured output of the system is the battery power, computed as the result of the difference between generated power and consumed power:

(27) $$y=P_{bat}=P_{net}=P_{PMSG}+P_{PV}-P_{Load}$$

It is assumed that the load power is known and considered as one of the system inputs.

#### 2.5.2 Load side

On the load side, equations (28), (29) define the state and output variables, respectively

(28) $$x_{Load}=\left[\begin{array}{cc} i_{L3} & v_{C3} \end{array}\right]$$

(29) $$y_{Load}=v_{C3}$$

The output variable described by equation (29) can correspond to either of the two states in the $x_{Load}$ vector defined by equation (28). However, for practical purposes, measuring voltage is more cost-effective and straightforward, as it does not require any modification to the circuit structure. In this study, the performance of SE algorithms on the load side is not investigated due to the similarity between the load model and the PV-array model. Therefore, only the states described by equation (26) (represented by vector x) are considered and utilized in the estimation process.

## 3 Unscented Kalman Filter

As previously mentioned, for nonlinear state estimation, the EKF is the most commonly chosen method in various applications. However, the performance of this algorithm is highly influenced by the degree of nonlinearity of the system at its operating point. Many systems are prone to sudden changes in their operating state or sudden step (or sometimes impulse) changes in input signals, which can have a notable impact on the accuracy and stability of the EKF. Employing various nonlinear transformations can enhance an algorithm's ability to handle such situations. These transformations form the foundation of the UKF, enabling the utilization of a wide range of nonlinear transformations to estimate nonlinear systems. These nonlinear transformations empower the UKF to effectively handle nonlinearities and sudden changes in the system's state of operation.

The Main idea behind the UKF was based on an intuition which says “it is easier to approximate a probability distribution than to approximate an arbitrary nonlinear function or transformation” [1]. In KF problems, it means that if we know x (i.e. its probability distribution), we can estimate the mean and covariance of y when we have y=g(x) and g(x) is a non-linear function of state variables.

The unscented Kalman filter (UKF) and the extended Kalman filter (EKF) differ in notable ways. In the EKF, the computation of the Jacobian matrix is necessary as it linearizes the nonlinear system. However, for an n-state system, computing the n^2^ elements of the Jacobian matrix can be computationally demanding. Nevertheless, when the Jacobian matrix is sparse due to minimal interdependence among state variables, its computation may not pose a significant challenge, thus obviating the need for Jacobian calculations. On the other hand, the UKF does not require the computation of a Jacobian matrix but instead utilizes the system's fundamental nonlinear equations. This eliminates the need for Jacobian calculations; however, the UKF does require 2n+1 computations for each time step. Moreover, in systems characterized by complex state equations, the computational demand of the UKF may be substantial.

There are various examples that advocate for the use of the UKF over the EKF in SE problems. For instance Ref. [46], demonstrates that EKF may diverge from the true state values, while the UKF consistently remains converged to the states. Additionally [47], shows that employing the UKF can reduce the mean error by more than 60 %. Furthermore, in the SE problem for an induction machine, the UKF used in Ref. [1] has a significantly lower error and a higher convergence rate compared to the EKF used in Ref. [48].

The estimation process begins with the propagation of sigma points, which are directly derived from a nonlinear transformation applied on the previously estimated states [49]. Each sigma point has its own weight and produces an output through the nonlinear function g. The covariance of the output y is then evaluated using the weighted outer product of the sigma point outputs. The following steps describe the main procedure of the UKF estimation algorithm for a discrete system with n states, represented by its state-space model as:

$$x_{k+1}=f\;\left(\;x_{k},u_{k},t_{k}\;\right)+w_{k}$$

(30) $$y_{k}=h\;\left({\;x}_{k},u_{k},t_{k}\;\right)+v_{k}$$

where w and v are process noise and measurement noise, respectively. The system described by equation (30) is a nonlinear system. It is assumed that the noises follow a Gaussian distribution and have a zero mean. Also, $Q_{k}$ is the covariance of process noise and $R_{k}$ is the covariance of measurement noise.

a) Initialization:

(31) $${\hat{x}}_{0}^{+}=E\;\left(x_{0}\right)$$

(32) $$P_{0}^{+}=E\;\left[\;\left(x_{0}-{\hat{x}}_{0}^{+}\right)\;{\left(x_{0}-{\hat{x}}_{0}^{+}\right)}^{T}\;\right]$$

b) Sigma points propagation

(33) $${\hat{x}}_{k-1}^{\left(i\right)}={\hat{x}}_{k-1}^{+}+{\tilde{x}}^{\left(i\right)}:i=1,2,\ldots ,2n$$

(34) $${\tilde{x}}^{\left(i\right)}={\left(\sqrt{\;{nP}_{k-1}^{+}}\right)}_{i}^{T}:i=1,\ldots ,n$$

(35) $${\tilde{x}}^{\left(i+n\right)}=-{\left(\sqrt{\;{nP}_{k-1}^{+}}\right)}_{i}^{T}:i=1,\ldots ,n$$

Note that $\sqrt{\;n\;P}$ is the matrix square root of the nP and ${\left(\sqrt{\;n\;P}\right)}_{i}$ is the i-th row of that matrix.

c) Sigma points transformation

The produced sigma points are transformed using the known non-linear function of the system to obtain their new states:

(36) $${\hat{x}}_{k}^{\left(i\right)}=f\;\left(\;{\hat{x}}_{k-1}^{\left(i\right)},u_{k},t_{k}\;\right)$$

d) *a priori* estimate at time k

The apriori estimate at this time instant is the mean of the transformed sigma points from the previous step (equation (36)):

(37) $${\hat{x}}_{k}^{-}=\frac{1}{2n}\;\sum_{i=1}^{2n}{\hat{x}}_{k}^{\left(i\right)}$$

Also, apriori error covariance is evaluated as well as $Q_{k-1}$ plays its role at this step:

(38) $$P_{k}^{-}=\frac{1}{2n}\;\sum_{i=1}^{2n}\left({\hat{x}}_{k}^{\left(i\right)}-{\hat{x}}_{k}^{-}\right)\;{\left({\hat{x}}_{k}^{\left(i\right)}-{\hat{x}}_{k}^{-}\right)}^{T}+Q_{k-1}$$

Consequently, the states are then updated from time instant k to time instant k−1.

e) Measurement sigma points propagation

(39) $${\hat{x}}_{k}^{\left(i\right)}={\hat{x}}_{k}^{-}+{\tilde{x}}^{\left(i\right)}:i=1,2,\ldots ,2n$$

(40) $${\tilde{x}}^{\left(i\right)}={\left(\sqrt{\;{nP}_{k}^{-}}\right)}_{i}^{T}:i=1,\ldots ,n$$

(41) $${\tilde{x}}^{\left(i+n\right)}=-{\left(\sqrt{\;{nP}_{k}^{-}}\right)}_{i}^{T}:i=1,\ldots ,n$$

It is important to note that we have the option to bypass this step and utilize the transformed sigma points from the time update section (equation (37)). While this approach reduces computational effort, it can potentially lead to increased errors.

f) Transform sigma points to measurement vectors

(42) $${\hat{y}}_{k}^{\left(i\right)}=h\;\left({\hat{x}}_{k}^{\left(i\right)},t_{k}\right)$$

Evaluating the mean of these measurement vectors will result in the predicted measurement vector:

(43) $${\hat{y}}_{k}=\frac{1}{2n}\;\sum_{i=1}^{2n}{\hat{y}}_{k}^{\left(i\right)}$$

In addition, considering $R_{k}$, we can estimate the error covariance of the predicted measurement vector:

(44) $$P_{y}=\frac{1}{2n}\;\sum_{i=1}^{2n}\left(\;{\hat{y}}_{k}^{\left(i\right)}-{\hat{y}}_{k}\right)\;{\left(\;{\hat{y}}_{k}^{\left(i\right)}-{\hat{y}}_{k}\right)}^{T}+R_{k}$$

g) Estimation of the cross covariance between ${\hat{x}}_{k}^{-}$ and ${\hat{y}}_{k}$ :

(45) $$P_{xy}=\frac{1}{2n}\;\sum_{i=1}^{2n}\left({\hat{x}}_{k}^{\left(i\right)}-{\hat{x}}_{k}^{-}\;\right)\;{\left(\;{\hat{y}}_{k}^{\left(i\right)}-{\hat{y}}_{k}\right)}^{T}\;$$

h) Kalman filter gain and estimation update:

(46) $$K_{k}=P_{xy}\;P_{y}^{-1}$$

(47) $${\hat{x}}_{k}^{+}={\hat{x}}_{k}^{-}+K_{k}\;\left(y_{k}-{\hat{y}}_{k}\right)$$

(48) $$P_{k}^{+}=P_{k}^{-}-K_{k}\;P_{y}\;K_{k}^{T}$$

**Fig. 5:**
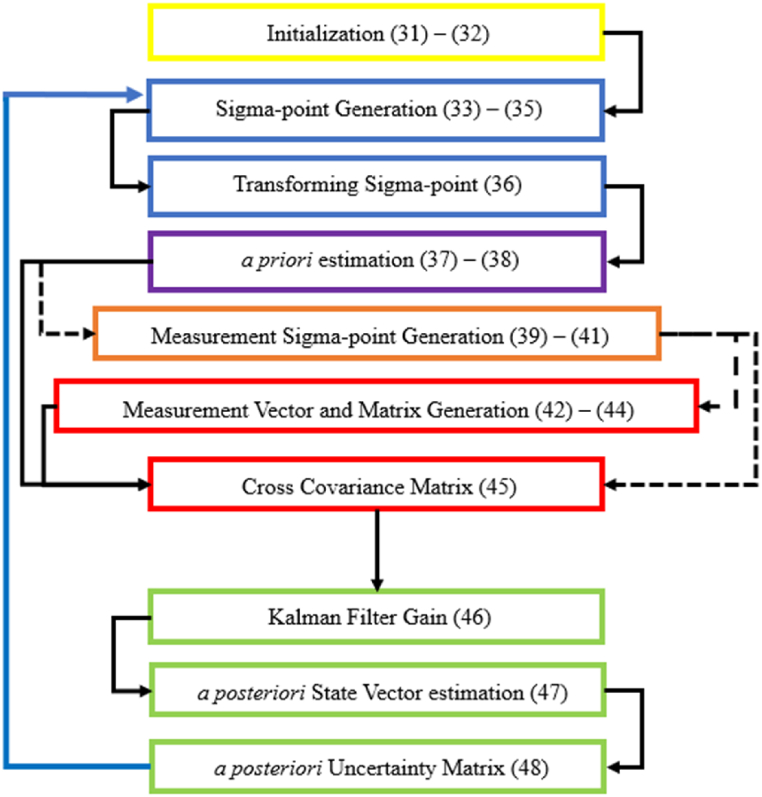
UKF flowchart.

Equations (47) and (48) serves as the initial values for the estimation process in the next step. This means that the ${\hat{x}}_{k}^{+}$ and $P_{k}^{+}$ obtained from this step will be used as ${\hat{x}}_{0}^{+}$ and $P_{0}^{+}$ in equations (31) and (32) for the next time instant (i.e. k+1). Fig. 5 summerizes these steps in a flowchart.

**Fig. 6:**
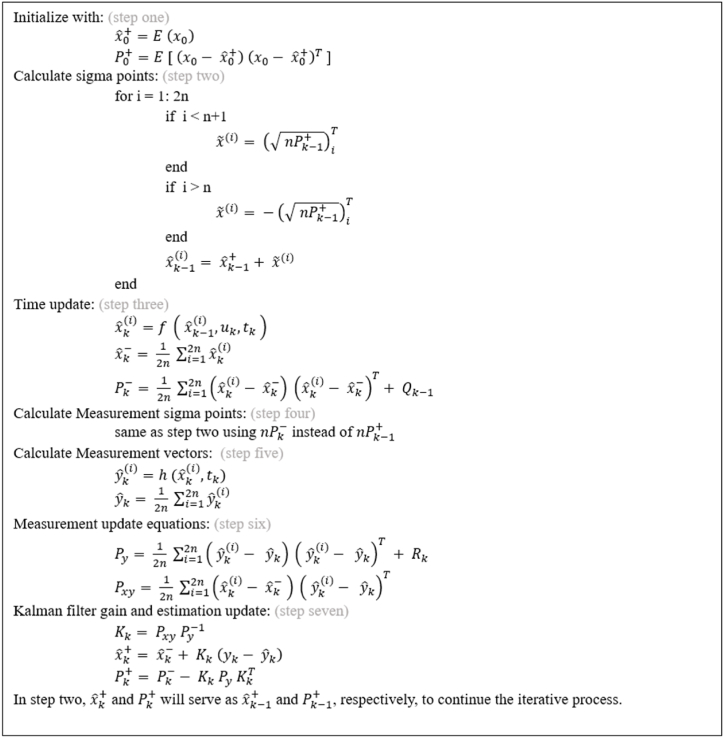
Pseudocode for unscented Kalman filter.

This flowchart can also be represented as a pseudocode as shown in Fig. 6.

It should be noted that in equations (34), (35), sigma points can be propagated by defining alternative coefficients for $P_{k-1}^{+}$. Additionally, all equations involving a summation (e.g., (38), (43) (44), etc.), can utilize a weighted sum instead. These modifications, as represented in Ref. [50], offer greater flexibility for algorithm tuning.

## 4 Implementation

**Fig. 7:**
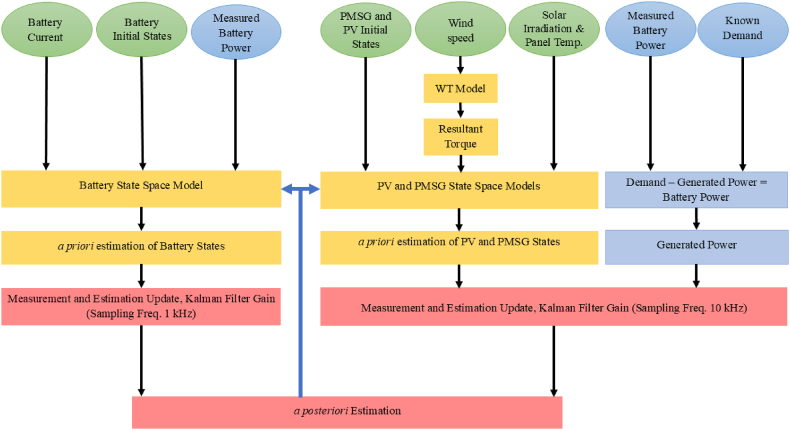
Implementation of UKF on the MG as an estimation algorithm.

The UKF estimation method described in the previous section can be implemented on the MG under study, as showed in Fig. 1. Additionally, Fig. 7 provides an overview of this implementation as an estimation algorithm, the output of which is a fundamental element for the EMS. It should be noted that this study focuses solely on the SE problem on the generation and storage side, as outlined in Section 2.5.1. The analysis of the estimation process on the load side is omitted for two reasons: first, its equations are similar to those derived for the boost converter; second, they are linear, resulting in a straightforward SE process. Moreover, the load side system is decoupled from the generation and storage system, allowing the estimation process to operate independently.

In the estimation process, as discussed for discrete systems in the previous section, the equations presented in the last section are continuous. These continuous equations can be converted into discrete equations using the basic definition of the time derivative, which is represented as follows:

(49) $$\dot{x}=\frac{x\left(k\right)-x\left(k-1\right)}{\Delta t}$$

By rearranging equations (49), (30), we can reform the state equations in discrete space:

$$x\left(k\right)=x\left(k-1\right)+\Delta t\;.\;f\;\left(x_{k-1},u_{k-1},w_{k-1}\right)$$

(50) $$y\left(k\right)=h\;\left(x_{k},u_{k},v_{k}\right)$$

Where Δt represents the sampling time.

As mentioned previously, the MG model comprises nine state variables, represented by equations (1), (2), (5), (9), (10), (14), (15). These equations define the function $f\;\left(\;x_{k},u_{k},t_{k}\;\right)$ in equation (30). Additionally, equation (27) represents the function $h\;\left({\;x}_{k},u_{k},t_{k}\;\right)$ in equation (30) and will be utilized in conjunction with the function $f\;\left(\;x_{k},u_{k},t_{k}\;\right)$ within the UKF algorithm applied for SE (i.e., equations (31), (32), (33), (34), (35), (36), (37), (38), (39), (40), (41), (42), (43), (44), (45), (46), (47), (48)). In the next step, this state-space model will be discretized using equation (50). The primary focus is on seven states (excluding battery voltage drops) and the output (or input) power of generation and storage units.

The estimation process starts with the initialization of the estimated states. This initialization is very important in the operation of the UKF as any divergence in the estimation of initial states can lead to an overall divergence of the estimation process. This initialization involves providing an initial guess for the states and their associated uncertainty matrix, $P_{0}^{+}$. The $P_{0}^{+}$ matrix represents the estimated accuracy of the state estimates and evolves over time according to the process dynamics and the received measurements. Ref. [51], provides a detailed description of the selection and tuning of the $P_{0}^{+}$ matrix.

**Fig. 8:**
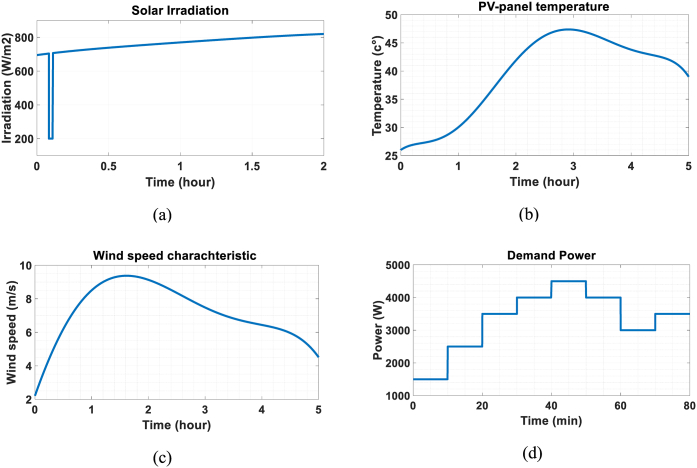
System inputs. a) solar irradiation, b) PV-panel temperature, c) wind speed and d) demand power.

In this estimation algorithm, the inputs of the system are known which are emphasized by circles in Fig. 7. System inputs, which include solar radiation, PV-cell temperature, wind speed, and demand power, are given as shown in Fig. 8(a)–8(d), respectively. Furthermore, according to equation (27), the battery power is assumed to be measured, and with the knowledge of the load (demand) power, the generated power of the RES units can be evaluated through the battery power. As depicted in Fig. 7, this allows us to divide the main estimation process into two independent and parallel processes. The measured power of the battery directly serves as the measurement signal for the first estimation process, while the generated power of RES units acts as the measurement signal for the second process. The objective of the later process is to estimate the states of the PV-array and the PMSG simultaneously. As previously mentioned, KF-based estimation methods are recursive, and thus, the output from each estimation serves as the initial for the next time instant, as shown by the upward arrows in Fig. 7.

### 4.1 Battery estimation process

As mentioned previously, the first process estimates the battery's SOC using its measured power signal, which is subject to noise. In this MG, there are thirty battery cells arranged into three parallel strings, with each string comprising ten cells connected in series. Consequently, the power capacity of each cell is $\frac{1}{30}$ of the total net power. The specifications of the cells are provided in Table 1 [37]. For the studied battery [37], a sixth-order relation (based on equation (6)) is established between the OCV and SOC as follows:

(51) $$U_{ocv}=f\left(SOC\right)=2.791+13.17\times SOC-64.79\times {SOC}^{2}+167.9\times {SOC}^{3}-235.2\times {SOC}^{4}+166.8\times {SOC}^{5}-46.57\times {SOC}^{6}$$

**Table 1:** Battery Cell Parameters [37].

Cell type	LiFePO4
Nominal Capacity	10 Ah
Nominal Voltage	3.6 V
$R_{o}$	38.57 mΩ
$R_{1}$	1.02 mΩ
$R_{2}$	0.35 mΩ
$C_{1}$	1.09 kF
$C_{2}$	42.09 kF
Coulomb efficiency	0.98

**Fig. 9:**
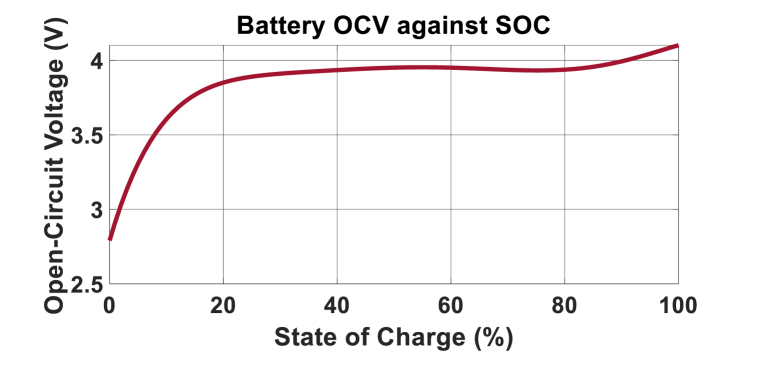
Variation of OCV against SOC.

Fig. 9 shows the behavior of the OCV with respect to the SOC. Furthermore, the voltage drops described by equations (1), (2) will be in millivolts, while the OCV is about 3–4 V for the standard state of operation (no over or under discharging). For simplicity and to reduce computational effort, the voltage drop across these RC branches can be neglected, although this sacrifices accuracy and precision. This approximation will be particularly helpful in cases where there are a large number of states in the system of interest.

As previously mentioned, each string of the storage system consists of 10 cells connected in series. Referring to Fig. 9, the output voltage of each parallel string, including voltage drops, ranges from 36 to 40 V across a wide range of SoC. Additionally, as shown in Fig. 1, a high-gain DC-DC boost converter is employed to step up this voltage to 360 V, which is the voltage of the common DC bus in the microgrid.

### 4.2 Generation units estimation process

The parameters of the wind turbine and PMSG are listed in Table 2, Table 3, respectively [43]. According to Table 2, the PMSG can operate at an output voltage of 220 V (line to neutral), which will be transformed to 360 V for the common DC bus of the microgrid. The state-space model parameters for the PV array are provided in Table 4 [39]. Additionally, this table indicates that the output voltage of the PV array will be approximately 320 V, which will be stepped up to 360 V for the common DC bus using a DC-DC boost converter. As previously demonstrated, the total generated power from the PMSG and PV units can be evaluted by measuring the load power and the input or output power of the battery.

**Table 2:** Wind turbine parameters.

Model	SENWEY SW-40	
Rotor diameter:	15.0 m	
Blades quantity:	3 pcs	
Direction:	upwind	
Rated output Power:	40.0 kW	
Maximum Power:	42.0 kW	
working voltage:	AC - 220V/380V	
working wind speed:	3–25 m/s	
Initial wind speed:	3.0 m/s	
Nominal wind speed:	12.0 m/s	
Storm-stand:	up to 50 m/s	
Wind turbine type:	3ph, PMG Alternator

**Table 3:** PMSG parameters [43].

Damping Coefficient	0.5
Rotor Inertia	13
$T_{d0}^{\prime }$	0.131 s
$T_{q0}^{\prime }$	0.0131 s
$x_{d}$	2.06
$x_{q}$	1.214
$x_{d}^{\prime }$	0.375
$x_{q}^{\prime }$	0.375

**Table 4:** PV-panel and converter parameters [39].

Nominal Power	5 KW
Nominal Voltage	326.6 V
Efficiency	0.97
Nominal Current	10.25 A
$I_{ph0}$	15.88 A
$I_{s0}$	744 pA
$a_{0}$	18.34 V
$R_{s0}$	2.55 Ω
$R_{sh0}$	531.5 Ω
$a_{I_{\text{sc}}}$	0.06 %
$C_{\text{pv}}$	470 μF
$L_{\text{dc}}$	0.6 mH
$R_{\text{dc}}$	0.3 Ω

## 5 Simulation and results

### 5.1 Estimation process of battery

**Fig. 10:**
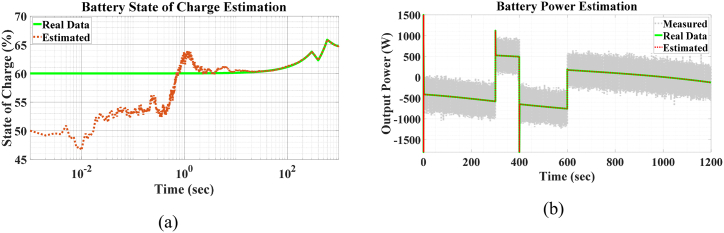
Estimation of a) battery SOC and b) battery output power.

For this estimation process, simulations were performed with a sampling time of Δt=1ms. The UKF algorithm may divegre with Δt=0.01s, espescially in the presence of sudden changes in demand power. This can lead to sudden changes in the output or input power of the battery, as storage systems are the components which handle the power balance. The estimation results are shown in Fig. 10(a) and (b). As it can be seen, the UKF algorithm can rapidly and accurately converge on the power of the battery. The error of this estimation is less than 0.1 percent. Fig. 10 (a) displays the estimated SOC of the battery, which takes slightly longer to converge due to a far initial guess. However, it eventually converges with a high degree of accuracy and remains stable, even in the presence of subsequent abrupt changes in load power. The covariance of the power signal noise is R=900 and it is assumed that the process is noise free, i.e., Q=0.

### 5.2 Estimation process of generation units

#### 5.2.1 Simulation

As previously mentioned, this process aims to estimate four states of the PMSG and two states of the PV-array. According to equations (31), (32), the estimation begins with an initial guess. Unlike the previous process, this estimation process requires a shorter sampling time of Δt=0.1ms. The UKF algorithm operates adequately with Δt=1ms for the PMSG but calls for a shorter sampling time for PV generation unit. Additionally, since both the PV-array and the PMSG undergo the estimation process simultaneously, the entire procedure is conducted at Δt=0.1ms. The measurement and process noise signals are similar to those employed in the previous estimation process. The $P_{0}^{+}$ matrix is selected to indicate how far the estimates are from the actual state values. This matrix and the initial estimation are determined as follows:

$${\hat{x}}_{0}^{+}=\left[\begin{array}{c} \begin{array}{c} {\hat{\delta }}_{0}^{+}\\ {\hat{\omega }}_{0}^{+} \end{array}\\ \begin{array}{c} {\hat{e_{q}^{'}}}_{0}^{+}\\ {\hat{e_{d}^{'}}}_{0}^{+} \end{array}\\ \begin{array}{c} {\hat{V_{pv}}}_{0}^{+}\\ {\hat{I_{L}}}_{0}^{+} \end{array} \end{array}\right]=\left[\begin{array}{c} \begin{array}{c} 0.02\\ 0\\ 0.3 \end{array}\\ \begin{array}{c} 0.3\\ 160\\ 20 \end{array} \end{array}\right]\;and\;\;P_{0}^{+}=\left[\begin{array}{cc} \begin{array}{ccc} \begin{array}{c} \begin{array}{c} 0.001\\ 0\\ 0 \end{array}\\ \begin{array}{c} 0\\ 0\\ 0 \end{array} \end{array} & \begin{array}{c} \begin{array}{c} 0\\ 0.01\\ 0 \end{array}\\ \begin{array}{c} 0\\ 0\\ 0 \end{array} \end{array} & \begin{array}{c} \begin{array}{c} 0\\ 0\\ 0.1 \end{array}\\ \begin{array}{c} 0\\ 0\\ 0 \end{array} \end{array} \end{array} & \begin{array}{ccc} \begin{array}{c} \begin{array}{c} 0\\ 0\\ 0 \end{array}\\ \begin{array}{c} 0.1\\ 0\\ 0 \end{array} \end{array} & \begin{array}{c} \begin{array}{c} 0\\ 0\\ 0 \end{array}\\ \begin{array}{c} 0\\ 100\\ 0 \end{array} \end{array} & \begin{array}{c} \begin{array}{c} 0\\ 0\\ 0 \end{array}\\ \begin{array}{c} 0\\ 0\\ 100 \end{array} \end{array} \end{array} \end{array}\right]$$

To examine the robustness of the algorithm, we intentionally misinitialize it by swapping the last two rows of the ${\hat{x}}_{0}^{+}$ vector and $P_{0}^{+}$ matrix. Consequently, ${\hat{x}}_{0}^{+}$ and $P_{0}^{+}$ will be as follows:

$${\hat{x}}_{0}^{+}=\left[\begin{array}{c} \begin{array}{c} {\hat{\delta }}_{0}^{+}\\ {\hat{\omega }}_{0}^{+} \end{array}\\ \begin{array}{c} {\hat{e_{q}^{'}}}_{0}^{+}\\ {\hat{e_{d}^{'}}}_{0}^{+} \end{array}\\ \begin{array}{c} {\hat{V_{pv}}}_{0}^{+}\\ {\hat{I_{L}}}_{0}^{+} \end{array} \end{array}\right]=\left[\begin{array}{c} \begin{array}{c} 0.02\\ 0\\ 0.3 \end{array}\\ \begin{array}{c} 0.3\\ 20\\ 160 \end{array} \end{array}\right]\;and\;\;P_{0}^{+}=\left[\begin{array}{cc} \begin{array}{ccc} \begin{array}{c} \begin{array}{c} 0.001\\ 0\\ 0 \end{array}\\ \begin{array}{c} 0\\ 0\\ 0 \end{array} \end{array} & \begin{array}{c} \begin{array}{c} 0\\ 0.01\\ 0 \end{array}\\ \begin{array}{c} 0\\ 0\\ 0 \end{array} \end{array} & \begin{array}{c} \begin{array}{c} 0\\ 0\\ 0.1 \end{array}\\ \begin{array}{c} 0\\ 0\\ 0 \end{array} \end{array} \end{array} & \begin{array}{ccc} \begin{array}{c} \begin{array}{c} 0\\ 0\\ 0 \end{array}\\ \begin{array}{c} 0.1\\ 0\\ 0 \end{array} \end{array} & \begin{array}{c} \begin{array}{c} 0\\ 0\\ 0 \end{array}\\ \begin{array}{c} 0\\ 100\\ 0 \end{array} \end{array} & \begin{array}{c} \begin{array}{c} 0\\ 0\\ 0 \end{array}\\ \begin{array}{c} 0\\ 0\\ 100 \end{array} \end{array} \end{array} \end{array}\right]$$

This alteration will challenge the algorithm because the $P_{0}^{+}$ matrix can no longer accurately represent the distance from the true states. As a result, this will affect the entire algorithm, as the states in the sigma points are propagated according to equations (33), (34), (35) which includes both $P_{0}^{+}$ and ${\hat{x}}_{0}^{+}$. A significant unknown deviation in one or two states may lead to substantial deviations in all estimated states.

#### 5.2.2 Results

**Fig. 11:**
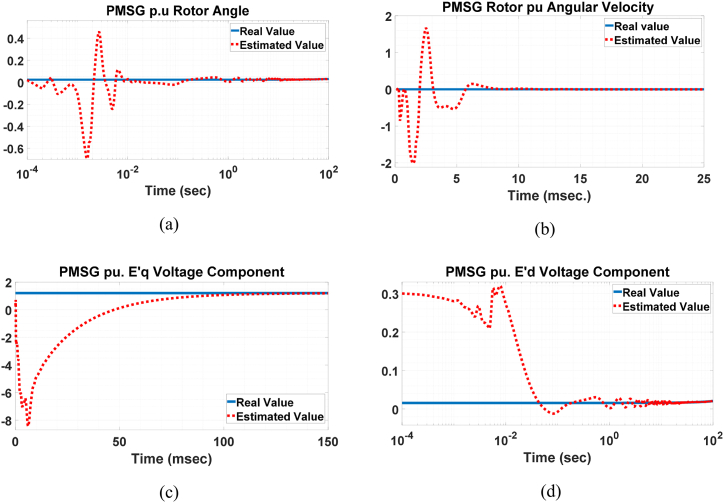
Estimation of PMSG a) Rotor angle, b) rotor angular velocity, c) q voltage component and d) d voltage component.

**Fig. 12:**
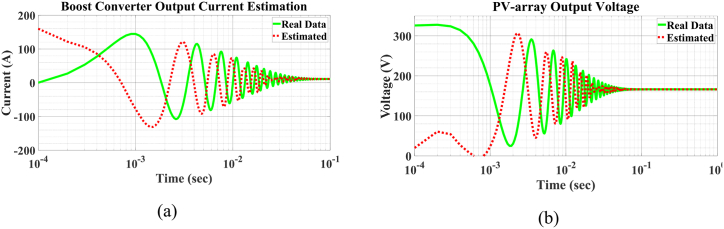
Initial estimation of a) converter inductor current and b) PV-array output voltage.

**Fig. 13:**
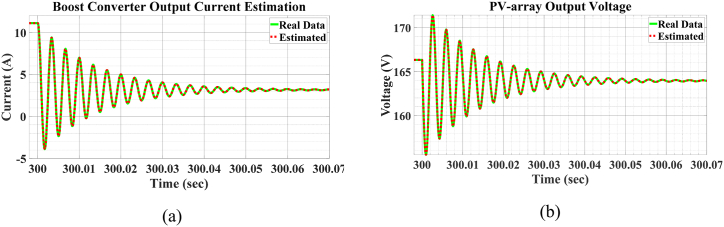
Estimation of a) converter inductor current and b) PV-array output voltage after sudden changes in irradiation.

**Fig. 14:**
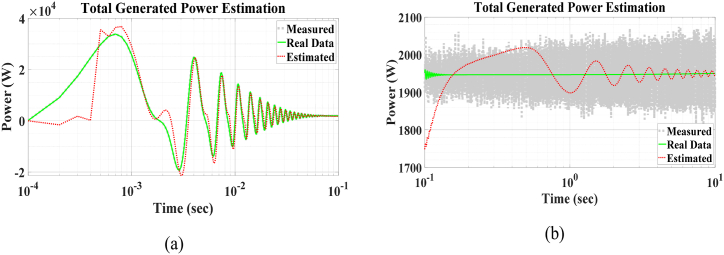
PV + WT generated power.

The results of this process are presented in Fig. 11, Fig. 12, Fig. 13, Fig. 14. In some of these figures, a logarithmic scale is used, as the process initially aims for convergence within the first few seconds or even milliseconds. This scale provides a clearer representation of the estimation process's progress. It is evident that our initial guess often deviates significantly from the true state value. Nevertheless, the UKF algorithm consistently converges to the true state value with commendable accuracy and speed. As shown in Fig. 11(c), a significant deviation from the actual value occurs from the beginnig until t=6.5ms. A similar deviation is observed in Fig. 11(a) and (b). The reason for this deviation is evident in Fig. 12(a) and (b), where the actual values are far from the estimates, but matrix $P_{0}^{+}$ cannot assist the algorithm in detecting this gap due to the earlier intentional swap. After t=1ms, the estimation of the two states in Fig. 12 begins to converge with greater accuracy and speed, resulting in the estimates in Fig. 11(a) and (c) approaching the actual state values. This increase in the rate of convergence is also evident in Fig. 11(d). Additionally, the robustness of the system is examined against a sudden change in one of the inputs, specifically solar irradiation. This change causes transient behavior in the boost converter. However, as shown in Fig. 13(a) and (b), the UKF algorithm remains well-converged despite these abrupt changes.

The results of the total generated power estimation are presented in Fig. 14(a) and (b). The transient behavior of the boost converter causes its transient power to differ significantly from its steady-state power, with the transient power being nearly 20 times larger. The results are shown in two separate time intervals: the first from the beginning to t=100ms, and the second from t=100ms to t=10s.

## 6 Conclusion

In this study, a robust estimation algorithm was applied to an isolated MG equipped with both solar and wind generation units and an energy storage system. To accurately model the various components within the MG, non-linear state-space models were developed. To address the non-linear nature of the processes, the unscented transformation was employed for unscented estimation. This transformation allowed for the rapid and accurate estimation of the system's states. To evaluate the robustness of the algorithm, various situations were tested, including incorrect initialization and sudden changes in system inputs. Convergence was achieved within a short timeframe, ranging from a few milliseconds for some states to slightly longer for others, while still maintaining an acceptable convergence rate. Although the convergence rate was initially affected by intentionally misinitialization, the algorithm ultimately converged to the true state values. Sudden changes in inputs were also present in both SOC estimation and state estimation of generation units. However, the UKF algorithm demonstrated resilience against such abrupt changes. Furthermore, by making specific assumptions about the estimation process and component modeling, the algorithm reduced the computational load, making it suitable for real-time estimation problems that require quick responses. For future work, since the outputs of such SE algorithms are the necessary inputs for EMSs, the performance of this algorithm can be explored in other scenarios, such as short circuits occurring in microgrids. Another avenue for further research involves detailed modeling of power conversion components, such as boost converters or AC/DC converters, to enhance their integration into state estimation algorithms.

## CRediT authorship contribution statement

**Nima HajiHeydari Varnoosfaderani:** Writing – original draft, Visualization, Validation, Software, Methodology, Investigation, Conceptualization. **Amir Khorsandi:** Writing – review & editing, Validation, Supervision.

## Data availability

No new data was generated for the research described in the article.

## Funding

There is no funding available for this article.

## Declaration of competing interest

The authors declare that they have no known competing financial interests or personal relationships that could have appeared to influence the work reported in this paper.
